# RETRACTED: Yen et al. *n*-Butylidenephthalide Regulated Tumor Stem Cell Genes EZH2/AXL and Reduced Its Migration and Invasion in Glioblastoma. *Int. J. Mol. Sci.* 2017, *18*, 372

**DOI:** 10.3390/ijms27146095

**Published:** 2026-07-08

**Authors:** Ssu-Yin Yen, Hong-Meng Chuang, Mao-Hsuan Huang, Shinn-Zong Lin, Tzyy-Wen Chiou, Horng-Jyh Harn

**Affiliations:** 1 Department of Life Science and Graduate Institute of Biotechnology, National Dong Hwa University, Hualien 974, Taiwan; momoyin911@gmail.com; 2 Buddhist Tzu Chi Bioinnovation Center, Tzu Chi Foundation, Hualien 970, Taiwan; kavin273@gmail.com (H.-M.C.); spleo0825@gmail.com (M.-H.H.); shinnzong@yahoo.com.tw (S.-Z.L.); 3 Department of Life Sciences, National Chung Hsing University, Taichung 402, Taiwan; 4 Department of Neurosurgery, Buddhist Tzu Chi General Hospital, Hualien 970, Taiwan; 5 Department of Pathology, Buddhist Tzu Chi General Hospital and Tzu Chi University, Hualien 970, Taiwan

The journal retracts the article titled, “*n*-Butylidenephthalide Regulated Tumor Stem Cell Genes EZH2/AXL and Reduced Its Migration and Invasion in Glioblastoma” [1], cited above.

Following publication, concerns were brought to the attention of the Editorial Office regarding the integrity of the data published in this article [1].

In accordance with the journal’s standard procedures, the Editorial Office and Editorial Board conducted an investigation that confirmed duplication within a panel presented in Figure 5. Additional concerns relating to data analysis were also identified. Although the authors cooperated with the investigation, the explanations and supporting materials provided were not deemed sufficient by the Editorial Board to resolve the identified concerns. As a result, the Editorial Board has lost confidence in the reliability of the findings reported in this article and has decided to retract the publication.

This retraction was approved by the Editor-in-Chief of the journal *IJMS*.

The authors have been informed of this retraction. Horng-Jyh Harn agrees to this retraction. Ssu-Yin Yen disagree to this retraction. The remaining authors did not provide a comment on this decision.
